# Characteristics of a pseudolysogenic phage vB_YpM_HQ103 infecting *Yersinia pestis*

**DOI:** 10.1016/j.virusres.2024.199395

**Published:** 2024-05-30

**Authors:** Zijian Wang, Jiao Yang, Lihua Yang, Youhong Zhong, Peng Wang

**Affiliations:** Yunnan Key Laboratory for Zoonosis Control and Prevention, Yunnan Institute for Endemic Disease Control and Prevention, Dali, 671000, China

**Keywords:** *Y. pestis* phage, Temperature sensitive, Infective characteristic, Receptor, OmpA

## Abstract

The plague, caused by *Yersinia pestis*, is a natural focal disease and the presence of *Y. pestis* in the environment is a critical ecological concern worldwide. The role of *Y. pestis* phages in the ecological life cycle of the plague is crucial. Previously, a temperature-sensitive phage named vB_YpM_HQ103 was isolated from plague foci in Yunnan province, China. Upon infecting the EV76 strain of *Y. pestis*, vB_YpM_HQ103 exhibits lysogenic behavior at 21 °C and lytic behavior at 37 °C. Various methods including continuous passage lysogenic tests, in vitro lysis tests, comparative genomic assays, fluorescence quantitative PCR and receptor identification tests were employed to demonstrate that the lysogenic life cycle of this phage is applicable to wild *Y. pestis* strains; its lysogeny is pseudolysogenic (carrying but not integrating), allowing it to replicate and proliferate within *Y. pestis*. Furthermore, we have identified the outer membrane protein OmpA of *Y. pestis* as the receptor for phage infection. In conclusion, our research provides insight into the characteristics and receptors of a novel *Y. pestis* phage infection with a pseudolysogenic cycle. The findings of this study enhance our understanding of *Y. pestis* phages and plague microecology, offering valuable insights for future studies on the conservation and genetic evolution of *Y. pestis* in nature.

## Highlights

• A new *Y. pestis* phage was isolated and identified.
• we identified a pseudolysogenic *Y. pestis* phage. The phage existed as a plasmid-like at 21 °C and could enter the lytic cycle to release offspring when the temperature increased to 37 °C.
• we discovered that the outer membrane protein OmpA on the surface of *Y. pestis* is one of the receptors for phage HQ103.

## 1 Introduction

Plague is a severe infectious disease caused by *Y. pestis*, characterized by strong infectivity, rapid transmission, and high mortality rate (Barbieri et al., 2020). Plague is considered a natural focal disease with three worldwide pandemics. To investigate its prevalence, resting state, and the persistence of *Y. pestis* in nature is the increasing scientific research hotspot. Previous studies have shown that *Y. pestis* can infect more than 200 mammal species (Jullien et al., 2021), and soil contaminated by this bacterium may serve as a source of infection for mammals (Richgels et al., 2016). Consequently, research on the microecology of *Y. pestis* in animals and soil has gained increasing interest. phages can infect bacteria, as the most abundant and diverse organisms on Earth, with estimated numbers as high as 10^31^ (Atanasova et al., 2015). phages can be found in the intestinal tracts of humans and animals, as well as in soil, oceans, and every explored biological community (Dion et al., 2020). The "arms race" between phages and bacteria is a core element in the ecology and evolution of microbial communities, which is the process of mutual adaptation and counter-adaptation between species (Hampton et al., 2020). While phages with a lytic life cycle need to lyse the host to produce progeny phages, bacteria evolve various defense mechanisms to resist phage infection, and phages also evolve strategies to efficiently infect hosts (Koskella and Brockhurst, 2014). From the perspective of plague microecology, this "arms race" between phages and bacteria not only contributes to the evolutionary dynamics of plague populations;, but it also determines the epidemic and resting states of the disease.

We found that *Y. pestis* phages were present in areas where *Y. pestis* bacteria existed, including soil, water, and mammals like mice, shrews, dogs, and cats in Yunnan Province (Yuan et al., 2020; Jin et al., 2022). vB_YpM_HQ103 (HQ103), a temperature-sensitive *Y. pestis* phage, was isolated from a cecal sample of *Apodemus chevrieri* in Heqing County, Yunnan Province, China (Yang et al., 2023). This phage has a dsDNA genome of 31,962 bp and belongs to the *Peduoviridae* family. Interestingly, at 21 °C, HQ103 displays a lysogenic pattern, while at 37 °C, it enters the lytic cycle (Yang et al., 2023). Herein, we conducted in-depth research on this phage, including three consecutive generations of lysogenic experiments, in vitro lysis experiments, comparative genomic analysis, fluorescence quantitative PCR, and receptor identification experiments. This study aimed to determine whether the lysogenicity of HQ103 at 21 °C is universal, which lysogenic mode it follows, and whether it can return to the lytic cycle at 37 °C after entering the lysogenic life cycle. Additionally, we also investigate the receptor that this phage uses to infect *Y. pestis*. This study may provide theoretical support for exploring the ecological control of plague foci and offer valuable insights for the study of plague microecology.

## 2 Materials and methods

### 2.1 phage HQ103 infecting wild *Y. pestis*

Nineteen representative strains of *Y. pestis*, isolated for various years, and sources in Yunnan province, China (Table 1), were selected to study the infection characteristics of phage HQ103. To verify the universality of lysogeny, three consecutive generations of lysogenic experiments were conducted. Briefly, phage HQ103 and *Y. pestis* were thoroughly mixed at a multiplicity of infection (MOI) of 100 and allowed to adsorb for 3–5 min. 10 μL of the mixture was spread on LB solid plate, and three zones were drawn. Primers were designed based on a specific sequence in the HQ103 genome (F: cggttttaatgcagttgcatgagt, R: cttggattttcacatagtgtcgga). Ten single colony were randomly selected as templates for colony PCR identification (Walch et al., 2016). Three positive clones were randomly selected and streaked across three zones on three solid plates. The second-generation lysogenic bacteria were obtained after incubation at 21 °C. After identification, 42 single colonies (14 per plate) were randomly selected as templates for lysogenic detection. Three consecutive generations of experiments were performed, and the positive rate for each generation was calculated. Statistical analysis was conducted using SPSS 25.0 software.

**Table 1:** Information of *Yersinia pestis* strains used in this study.

Number	Strain number	Areas of separation	Years	Source of separation	Biotype	Genotype (Shi et al., 2021)
1	HQ16	Heqing County, Yunnan Province, China isolate	2017	Eothenomys miletus	Classical type	1.IN5
2	HQ32	Heqing County, Yunnan Province, China isolate	2017	Eothenomys miletus	Classical type	1.IN5
3	779	Jianchuan County, Yunnan Province, China isolate	1983	Neopsylla tenella	Classical type	1.IN3
4	1413	Jianchuan County, Yunnan Province, China isolate	1989	Eothenomys miletus	Classical type	1.IN3
5	LJ2	Yulong County, Yunnan Province, China isolate	2022	Rattus flavipectus	Classical type	1.IN5
6	LJ6	Yulong County, Yunnan Province, China isolate	2022	Eothenomys miletus	Classical type	1.IN5
7	488	Ruili City, Yunnan Province, China isolate	1982	Xenopsylla cheopis	Eastern Type	1.ORI2
8	2552	Yingjiang County, Yunnan Province, China isolate	2000	Xenopsylla cheopis	Eastern Type	1.ORI2
9	2037	Linxiang County, Yunnan Province, China isolate	1993	Xenopsylla cheopis	Eastern Type	1.ORI2
10	2441	Yun County, Yunnan Province, China isolate	1999	Rattus flavipectus	Eastern Type	1.ORI2
11	2167	Jinggu County, Yunnan Province, China isolate	1995	Rattus nitidus	Eastern Type	1.ORI2
12	2237	Mojiang County, Yunnan Province, China isolate	1996	Xenopsylla cheopis	Eastern Type	1.ORI2
13	2299	Wenshan County, Yunnan Province, China isolate	1996	Patient	Eastern Type	1.ORI2
14	2575	Wenshan County, Yunnan Province, China isolate	2002	Patient	Eastern Type	1.ORI2
15	2445	Menghai County, Yunnan Province, China isolate	2000	Rat (unknown)	Eastern Type	1.ORI2
16	MH38	Menghai County, Yunnan Province, China isolate	2020	Rattus flavipectus	Eastern Type	1.ORI2
17	1869	Yuanjiang County, Yunnan Province, China isolate	1992	Rattus flavipectus	Eastern Type	1.ORI2
18	2106	Tengchong County, Yunnan Province, China isolate	1994	Frontopsylla spadix	Eastern Type	1.ORI2
19	2290	Jianshui County, Yunnan Province, China isolate	1996	Rattus flavipectus	Eastern Type	1.ORI2

### 2.2 In vitro lysis of lysogenic bacteria

**Fig. 1:**
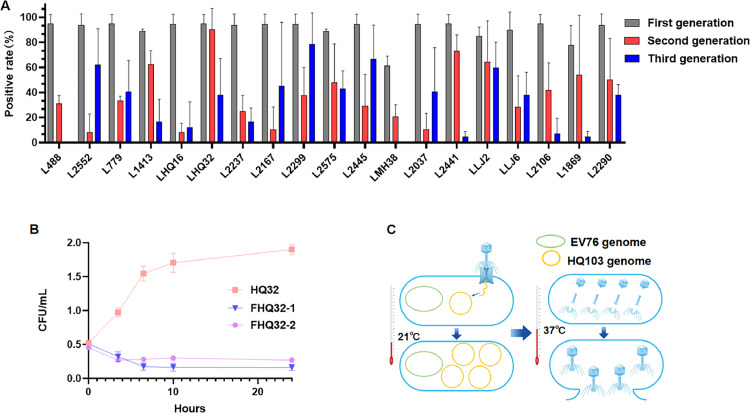
**Lysogenic assay and in vitro lysis assay. A**, Positive rate of three generations of lysogens of phage HQ103 infected 19 strains of *Y. pestis* in Yunnan Province. **B**, In vitro lysis experiments of wild and lysogenic strains of *Y. pestis*. **C**, phage invaded wild bacteria and transformed into plasmid and existed in the form of pseudolysogen at 21 °C, phage recovered lysis activity at 37 °C, and the progeny of phage were synthesized in bacteria again and released in vitro.

The lysogenic strain of wild *Y. pestis* HQ32 was selected for in vitro lysis to demonstrate whether that the phage in the pseudolysogenic state could be reintroduced to the lysis cycle. Two first-generation positive clones (FHQ32–1 and FHQ32–2) were selected and cultured in 1 mL LB liquid medium, with the wild-type HQ32 strain used as the control. The initial concentration of each group was adjusted to 0.5 McFarland, and then cultured in a 37 °Cshaking incubator at 220 rpm. The McFarland concentration was measured at regular intervals (Fig 1B), and the growth curve of lysogenic bacteria and wild strain at 37 °C was compared to determine whether HQ103 in lysogenic bacteria entered the lytic cycle.

### 2.3 Phage HQ103 in *Y. pestis*

To detect the existence status of phage HQ103 genome in lysogenic *Y. pestis*, whether it is a true lysogenic state integrated into the genome of *Y. pestis*, or a pseudolysogenic state carrying a plasmid and not integrated, four wild *Y. pestis* strains with different lysogenically positive rates and their lysogenic strains (phage HQ103 lysogenic *Y. pestis* 2299, 2575, 2445, and MH38, respectively; named as L2299, L2575, L2445, and LMH38, respectively) were used. DNA was extracted and sequenced. After data assembly, Bandage (Wick et al., 2015) was used for visualization, and pairwise comparison was carried out through BLAST.

### 2.4 Genetic detection of phage HQ103 genome in *Y. pestis*

To determine if the phage HQ103 genome in *Y. pestis* can replicate and transmitted to subsequent generations, we employed the SYBR Green dye method for relative quantification of phage HQ103 in the *Y. pestis* vaccine strain EV76.

16 s gene of *Y. pestis* was used as the reference gene for qPCR (16S-F: aagggcacaacctccaagt, 16S-R: tgtagcggtgaaatgcgtag), and the phage HQ103 replication protein gene *RPB* served as the target gene for detecting phage copy amounts (RPB-F: gcagatattgctctggttttac, RPB-R: gaattcaatcagccgtactgg). The first-generation (eight single colonies) and second-generation (24 single colonies) lysogenic bacteria were detected by qPCR. Briefly, a total of 40 cycles, including predenaturation at 95 °C for 2 min, denaturation at 95 °C for 10 s, annealing at 52 °C for 10 s, and extension at 72 °C for 20 s, were performed. Finally, the relative copy quantity of phage HQ103 in the first and second-generation lysogens was calculated, and compared methods (Schmittgen and Livak, 2008):

ΔCt = Ct _RPB_ - Ct 16 s, where Ct _RPB_ represents the Ct value of the RPB gene of phage HQ103 replication protein gene, Ct 16 s represents the Ct value of the 16 s gene of EV76 strain, and ΔCt represents the relative Ct value of phage HQ103 after excluding the internal control.

N_x_ = 2^−ΔCt^, where N_1_ represents the copy number of phage HQ103 relative to *Y. pestis* EV76 in the first-generation lysogens, and N_2_ represents the copy number of phage HQ103 relative to *Y. pestis* EV76 in the second-generation lysogens.

ΔΔCt = ΔCt_2_ - ΔCt_1_, ΔΔCt represents the actual difference in Ct values of the RPB gene, which is calculated by subtracting the internal control from the relative Ct values of phage HQ103 in the second and first-generation lysogens.

*M* = 2^−ΔΔCt^, M denotes the fold change in the copy number of phage HQ103 between the second and first-generation lysogens.

### 2.5 Identification of phage HQ103 receptor

To investigate the receptor utilized by phage HQ103 for adsorption, binding, and genome injection into the host, we conducted a study on the phage HQ103 receptor. Initially, we co-cultured high-titer phage HQ103 with *Y. pestis* EV76 in its logarithmic phase using the double plate method for 3–5 days. Once single colonies shown on the plate, we established the phage-resistant mutant EV76R through three successive passages. Subsequently, we sequenced both EV76 and EV76R to identify the mutation sites in the resistant mutants.

A complementation test was performed for the mutant OmpA gene. PCR amplification of the OmpA gene was carried out using primers OmpA-F (gagggaaaattatgtctagaatgagtcggtggtggcaac) and OmpA-R (Ctaaaaacctacagaagcttttacttctccatttcttgcgttgatacgt). The linear plasmid PLI50–10 s was amplified using primers PLI50–10s-HQ103-F (Cgcaagaaatggagaagtaaaagcttctgtaggtttttaggcataaaactatatgatttacc) and PLI50–10s-HQ103-R (Cgttgccaccaccgactcattctagacataattttccctccttattcgtct). The purified PCR products were then assembled using the Gibson assembly cloning method to generate the recombinant plasmid PLI-OmpA. The plasmid PLI-OmpA was extracted using the Omega Bio-tek plasmid extraction kit and verified by Sanger sequencing. The recombinant plasmid was transformed into competent cells of the EV76 resistant mutant EV76R via electroporation, generating the EV76R complement strain. Successful single colony were screened on an LB solid medium containing ampicillin (100 µg/mL).

Ultimately, the adsorption rate and plaque formation rate of phage HQ103 were tested on EV76, mutant EV76R, and complement strain EV76R::OmpA to confirm whether OmpA served as the binding receptor for HQ103 and EV76.

## 3 Results

### 3.1 phage HQ103 infecting wild *Y. pestis*

19 strains of *Y. pestis* from diverse sources in Yunnan Province, China, were infected with phage HQ103. PCR results and agarose gel electrophoresis revealed that all 19 *Y. pestis* strains were susceptible to infection by phage HQ103. The positive rate of lysogenic bacteria in the other strains was greater than or equal to 80%, and the positive rate of 12 strains reached 100%, except for 2 strains (55.56% positive rate of FMH38 and 66.67% positive rate of F1869) (Fig. 1A) .

Three clones were randomly selected from the first-generation positive single colony and passaged to obtain second-generation strains. The results revealed that the second-generation positive rates for strains S1413, S1869, S2441, SHQ32, S2290, and SLJ2 were all equal to or greater than 50 %. Notably, the second-generation positive rates for SHQ32 and SHQ16, isolated from the same location are significantly different. The lysogens' positive rate in the former was 90.48%, while in the latter, it was as low as 8.3%. These results indicated that there was no correlation between the positive rate of lysogen and the region (Tables 2 and 3).

**Table 2:** Second-generation positive rates.

Strain number	Clone 1	Clone 2	Clone 3	Total positive rate
488	25 %	37.50 %	31.25 %	31.25 %
2552	0	0	25 %	8.30 %
779	37.50 %	31.25 %	31.25 %	33.33 %
1413	68.75 %	50.00 %	68.75 %	62.50 %
2237	12.50 %	37.50 %	25 %	25 %
2167	31.25 %	0	0	10.41 %
1869	75 %	87.50 %	0 %	54.12 %
2037	25 %	0.00 %	6.25 %	10.40 %
2441	62.50 %	68.75 %	87.50 %	75 %
16	12.50 %	12.50 %	0	8.30 %
2106	43.75 %	62.50 %	18.75 %	41.67 %
2299	18.75 %	31.25 %	62.50 %	37.50 %
2575	68.75 %	62.50 %	12.50 %	47.91 %
2290	87.50 %	37.50 %	25 %	50 %
2445	43.75 %	0	43.75 %	29.17 %
MH38	18.75 %	12.50 %	31.25 %	20.83 %

**Table 3:** Third-generation positive rates.

Strain number	Clone 1	Clone 2	Clone 3	Total positive rate
488	0.00 %	0.00 %	0.00 %	0.00 %
2552	92.86 %	35.71 %	57.14 %	61.90 %
779	14.28 %	42.86 %	64.26 %	40.48 %
1413	0.00 %	35.71 %	14.29 %	16.67 %
2237	28.57 %	7.14 %	14.28 %	16.67 %
2167	100.00 %	0.00 %	35.71 %	45.24 %
1869	7.14 %	7.14 %	0.00 %	5.00 %
2037	57.14 %	64.24 %	0.00 %	40.48 %
2441	7.14 %	7.14 %	0.00 %	4.76 %
16	0.00 %	35.71 %	0.00 %	11.90 %
2106	0.00 %	0.00 %	21.43 %	7.14 %
2299	50.00 %	92.86 %	92.86 %	78.57 %
2575	42.86 %	57.14 %	28.57 %	42.86 %
2290	28.57 %	42.86 %	42.86 %	38.10 %
2445	85.71 %	78.57 %	35.71 %	66.67 %
MH38	0.00 %	0.00 %	0.00 %	0.00 %

Three clones were randomly selected from the second-generation positive single colony and passaged to obtain the third-generation strains. The results showed that only strains T2552, T2299, T2445 and TLJ2 had a positive rate of more than or equal to 50 % in the third generation. Among the remaining strains, the positive rate of 5 strains was less than 10 %, and even the positive rate of T488 and TMH38 was as low as 0 %. The strains from the same area, such as T2299 and T2575 from Wenshan County and T2445 and TMH38 from Menghai County, showed significant differences. Statistical analysis results revealed significant differences between T2299 and T2575 (χ2=11.230, *p* < 0.05) and between T2445 and TMH38 in Menghai County (χ^2^=42.000, *p* < 0.05).

### 3.2 Lysogenic bacteria lysis

The McFarland concentration of the two selected lysogenic clones decreased to half starting from the 7th hour and remained stable. In contrast, the McFarland concentration of the wild strain increased logarithmically from 1 to 10 h and reached a plateau after 10 h (Fig. 1B) . Taken together, these results suggest that HQ103 in the lysogenic strain could be "activated" to enter the fission cycle at 37 °C, thereby steadily reducing the concentration of *Y. pestis (*Fig. 1C) .

### 3.3 phage HQ103 in *Y. pestis*

**Fig. 2:**
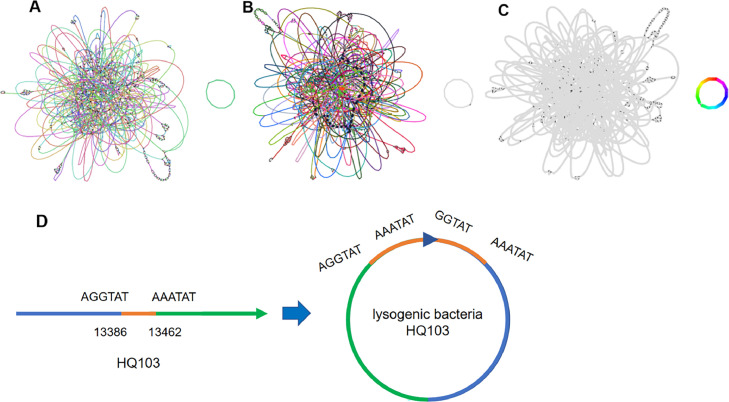
**Studies on the presence of phage HQ103 in *Y. pestis*. A**, Genome visualization of Lysogen L2299. **B**, Comparison of lysogen L2299 with primitive wild bacteria 2299. **C**, Comparison of Lysogen L2299 with phage HQ103.**D**,The original phage genome and the lysogenic state phage in bacteria.

The genome of lysogenic strain L2299 was compared to the wild strain of *Y. pestis*. The results showed that only the part on the left side of Fig 2B aligned, which was identified as the genome of *Y. pestis* 2299. A comparison and analysis of the lysogenic L2299 genome and phage HQ103 genome revealed that only the circular structure on the right side of Fig 2C completely matched, suggesting that this structure represents the phage HQ103 genome in lysogenic bacteria. Lysogenic strains L2445, L2575, and LMH38 displayed same results.

In this study, we extracted the genome sequences of phage HQ103 from two lysogenic strains, L2299 and L2575, collected from Wenshan County, Wenshan Prefecture. Compared to the original phage HQ103, 77 bases were added, and the genome was inverted. The phage genome from 1 to 18,577 bp in lysogens corresponded to the 13,386–31,962 bp of the original phage HQ103, and 18,578–32,039 bp matched the 1–13,462 bp of the original phage HQ103 (Fig 2D). We also extracted the genome sequence of phage HQ103 from two lysogenic strains (L2445 and LMH38) in Menghai County, Xishuangbanna Prefecture, and compared with original phage HQ103. We discovered that 77 bases were increased, and the genome was inverted. The 1–18,655 bp of the phage genome in lysogens corresponded to the 13,308–31,962 bp of the original phage HQ103, and the 18,656–32,039 bp matched the 1–13,384 bp of the original phage HQ103. A BLAST analysis of the 77 additional bases of phage HQ103 in lysogenic bacteria L2299 revealed that the sequence was a portion of the phage tail filament protein, and a comparison with the original HQ103 revealed that the sequence matched the 13,386–13,462 bp sequence precisely. We hypothesized that the 77 bp repeat sequence resulted from splicing errors. Consequently, a complete genome of phage HQ103 exists in lysogenic bacteria, and a ring structure is formed by the inversion of the genome to exist more stable.

The sequencing depth of *Y. pestis* strains 2299, 2445, 2575, and MH38 within the genomes of four lysogenic strains was approximately 100x. However, the sequencing depth of phage HQ103 in the genomes of these four lysogenic strains are different. In the genome of lysogenic strain L2299, the sequencing depth of phage HQ103 was 732x. In the genomes of lysogenic strains L2445, L2575, and LMH38, the sequencing depths of phage HQ103 were 1516x, 518x, and 1570x, respectively. These findings suggest that the copy number of phage HQ103 in lysogenic bacteria is not constant, with an average of 5–15 phages present in each bacterium.

### 3.4 Detection of phage HQ103 genome in *Y. pestis*

**Fig. 3:**
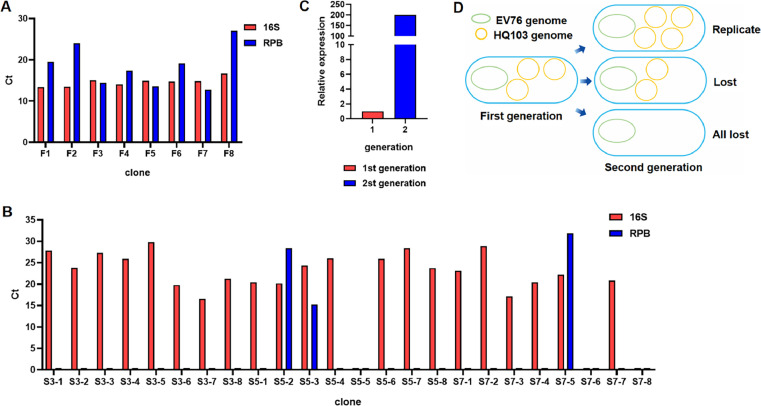
**Genetic detection of phage HQ103 genome in *Y. pestis*. A** and **B**, Represents the Ct values of the primers corresponding to phage and host bacteria in different single colony of the first and second generation lysogens respectively. The abscissa of A and B represent different single colony. In figure B, the second generation lysogen S3–1 represents the clone subcultured from the first generation lysogen F3 in figure A, and the ordinate represents the Ct values of two pairs of primers corresponding to each clone. **C**, Relative expression of phages in second-generation and first-generation lysogens. **D**, Schematic diagram of replication and loss of phages in lysogens with passage.

Three clones (F3, F5, and F7) from the first-generation lysogenic bacteria showed Ct _RPB_ < Ct 16 s (Fig 3A), indicating that the copy number of phage HQ103 in clones F3, F5, and F7 was higher than that of *Y. pestis* EV76. In contrast, the other five clones (F1, F2, F4, F6, and F8) exhibited Ct _RPB_ > Ct 16 s, suggesting that the copy number of phage HQ103 in these five clones was lower than that of *Y. pestis* EV76.

Among the 24 clones selected from the second-generation lysogenic bacteria, only clone S5–3 displayed Ct _RPB_ < Ct 16 s. Clone S5–3 of the second-generation lysogenic strain was derived from clone F5 of the first-generation lysogenic strain. The ΔCt _(RPB-_16 s_)_ of clone S5–3 was smaller than that of clone F5, with N_1_=2.53, indicating that the copy number of phage HQ103 in the first-generation lysogenic strain was 2.53 times that of *Y. pestis* EV76. For N_2_=506, the ΔCT _(RPB-16S)_ of clone S5–3 was smaller than that of clone F5. The results demonstrated that the copy number of phage HQ103 in the second-generation lysogens was 506 times that of *Y. pestis* EV76. The copy number of phage HQ103 in the second-generation lysogenic clone S5–3 was higher than that in the first-generation lysogenic clone F5, *M* = 200, indicating that the number of phage HQ103 in the second-generation *Y. pestis* clone S5–3 was 200 times higher than the first-generation lysogenic clone F5 (Fig 3C). These findings suggests that phages in lysogenic bacteria can replicate themselves during passage.

In the second generation of lysogens, phage genes were not detected in 21 clones out of 23 clones, while 2 clones, S5–2 and S7–5, had detectable phage genes, but Ct _RPB_= > Ct 16 s. Clone S5–2 was generated from clone F5, and clone S7–5 was generated from clone F7. These results indicated that the copy number of phage HQ103 in the second-generation lysogenic clones S5–2 and S7–5 was lower than that in the first-generation lysogenic clones F5 and F7. This suggests that phages in lysogenic bacteria are lost during passage. Based on these findings, we hypothesize that phages in lysogenic bacteria enter offspring bacteria asymmetrically during bacterial binary division. Phage replication and loss occur during bacterial growth and passage (Fig. 3D), which also supports the hypothesis that the positive rate of the second and third-generation lysogen experiments was higher or lower in the offspring than in the previous generation (Table 4, Table 5).

**Table 4:** Ct values of two primer pairs for eight clones of first-generation lysogens.

Strain number	Ct _16s_	Ct _RPB_	ΔCt (RPB-16S)
F1	13.366	19.542	6.176
F2	13.46	24.038	10.578
F3	15.026	14.392	−0.634
F4	14.046	17.359	3.313
F5	14.889	13.55	−1.339
F6	14.75	19.137	4.387
F7	14.85	12.756	−2.094
F8	16.676	27.012	10.336

**Table 5:** Ct values of two primer pairs for eight clones of second-generation lysogens.

Strain number	Ct _16s_	Ct _RPB_	ΔCt (RPB-16S)
S3–1	27.757		
S3–2	23.82		
S3–3	27.299		
S3–4	25.874		
S3–5	29.802		
S3–6	19.772		
S3–7	16.572		
S3–8	21.18		
S5–1	20.475		
S5–2	20.181	28.427	8.246
S5–3	24.281	15.298	−8.983
S5–4	26.023		
S5–5			
S5–6	25.938		
S5–7	28.399		
S5–8	23.777		
S7–1	23.098		
S7–2	28.897		
S7–3	17.114		
S7–4	20.411		
S7–5	22.264	31.85	
S7–6			
S7–7	20.856		
S7–8			

note: (No values in the margins).

### 3.5 Receptor of phage HQ103

Discovery by comparison in the mutant strain, there were two amino acid changes in the OmpA gene. The 767 bp C changed to T, causing amino acid Ala-to change to Asp, and the 1154 bp G changed to A, causing amino acid Arg-to change to His. Using the prediction model of Protter (Omasits et al., 2014), we found that there is one intra-membrane domain, one outer membrane domain, three transmembrane domains, and one short chain within the membrane. The two amino acid mutations are located in the inner and outer membrane domains, so we hypothesize that these two mutations might have separate roles.

**Fig. 4:**
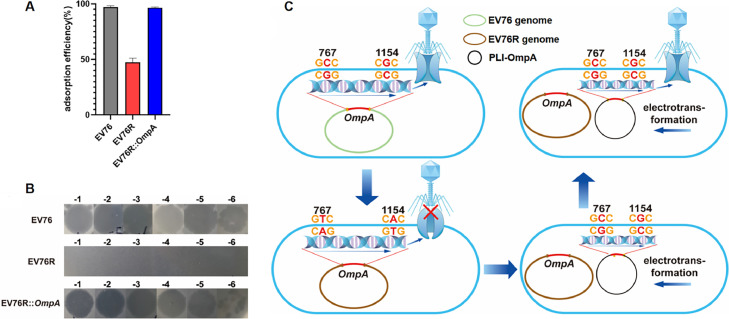
**Phage HQ103 receptor identification. A**, Adsorption rate experiments of wild strain, mutant strain and complementation strain. **B**, EOP experiments of phage HQ103 against wild strain, phage resistant strain EV76R and complementation strain EV76R::OmpA. **C**, Schematic diagram of receptor mutation and complementation process. The mutation of C at base 767 of OmpA gene in EV76 wild strain resulted in the change of amino acid Ala-to Asp, and the mutation of G at base 1154 resulted in the change of amino acid Arg-to His. The physicochemical properties of OmpA protein receptor were changed, so that the phage could not adsorb and bind. The unmutated OmpA gene was electroporated into the mutant strain. The phage can readsorb and bind to the replenishment strain.

The original OmpA gene was introduced into the mutant strain by constructing a complementation plasmid. Our results showed that the complemented strain returned to the wild type state, and the complemented strain could be lysed by the phage. The adsorption rate experiment (Fig. 4A) showed that the adsorption rate of phage HQ103 to the wild strain EV76 reached 97%, while the adsorption rate of the mutant strain EV76R was 47%, and the adsorption rate of the complemented strain EV76R::OmpA returned to the wild strain level. The plaque formation rate experiment (Fig 4B) showed that phage HQ103 infected the wild strain EV76, mutant strain EV76R, and complement strain EV76R::OmpA. The complement strain had an Efficiency of plating (EOP) consistent with the wild strain, while the mutant strain EV76R had no plaque, indicating that OmpA on *Y. pestis* was the receptor of *Y. pestis* phage HQ103.

## 4 Discussion

### 4.1 Phage HQ103 infection characteristics and biological significance in *Y. pestis*

Koskella et al. (Koskella and Brockhurst, 2014) posited that the coevolution between bacteria and phages plays a crucial role in driving the ecological and evolutionary processes of microbial communities. Hampton et al. (Hampton et al., 2020). reported that the infection pressure exerted by phages on bacteria can result in the development of various bacterial immune systems, corresponding to different stages of the phage life cycle. phages have also developed numerous strategies to counteract these defenses. Gregory et al. (Gregory et al., 2019) found that phages transfer approximately 10^^29^ genes between themselves and bacteria daily, enabling rapid evolution. Temperate phages integrate into the host genome via the lysogenic cycle and replicate alongside the host genome. This integration of phages into the host genome has a substantial impact on the biological phenotype and environmental adaptation of bacteria (Wang and Wood, 2016). Brussow et al. (Brüssow et al., 2004). discovered that phages encode many virulence factors. Bacteria such as Vibrio cholerae, Shiga toxin-producing Escherichia coli, Corynebacterium diphtheriae, and Clostridium botulinum depend on specific phage-encoded toxins to cause particular diseases. Another less common type of phage, filamentous phages, can multiply without causing the host bacteria to break apart, and these phages can also greatly impact the bacterial phenotype (Marston et al., 2012). Łoś et al. (Łoś and Węgrzyn, 2012) studied this unique phage life cycle called pseudolysogeny, which typically occurs due to unfavorable living conditions (like starvation) for phages, and when conditions improve, they re-enter the lytic cycle. However, Łoś et al. believed that phages in a pseudolysogenic state would not undergo genomic proliferation, which is contrary to our results. Moreover, most current reports on pseudolysogeny involve induction by mitomycin C to trigger lytic conversion (Geng et al., 2018), but our study shows that temperature can regulate the conversion from pseudolysogenic to lytic state. Consistent with our findings, Mantynen et al. (Mäntynen et al., 2021) reported that during bacterial proliferation, the phage genome in a pseudolysogenic state would be unevenly passed on to the offspring bacteria.

In this study, we identified a pseudolysogenic *Y. pestis* phage. The phage existed as a plasmid-like at 21 °C and could enter the lytic cycle to release offspring when the temperature increased to 37 °C. This phage was used to infect 19 strains of *Y. pestis* collected from 10 cities in Yunnan province, China. Our results showed that all 19 strains of *Y. pestis* could be lysogenized by phage HQ103, and the rate of lysogenization was not related to the place of isolation. During subculture, there are two situations, phage loss and replication. The differences in strains lead to different phage loss rates among strains. In lab experiments, lysogenic bacteria at 37 °C could effectively stop the growth of *Y. pestis* and keep its concentration low. When we detected the lysogenic genome, we found that the phage genome did not integrate into the chromosome but existed as plasmid-like. Real-time PCR showed that the phage-formed plasmid can replicate in Y. pestis. The amount of phage HQ103 in the second generation of Y. pestis was 200 times greater than in the first generation. The sequencing depth of the *Y. pestis* genome was about 100x, while the phage genome's sequencing depth was between 500 and 1500x, suggesting that there were 5–15 phages in each bacterium during the first generation of lysogens. However, Real-time PCR showed that the number of phages HQ103 copies in the first generation of lysogens was 2.2 times greater than in *Y. pestis* EV76. The data from fluorescence quantitative PCR may differ from the results of the other method in that it is relatively quantitative. At that time, it can only represent the condition of a particular colony. Some bacteria in a single colony may lack phages, so the relative quantitative data obtained is less significant than sequencing depth data. Ripp et al. proposed that pseudolysogens are important for keeping phage genetic material in ecosystems for a long time and helping phages survive in challenging environments. Pseudolysogenic phage genomes are found inside cells, which can protect phages from harsh conditions like ultraviolet rays, extreme pH levels, and temperatures (Ripp and Miller, 1997; Ripp and Miller, 1998; Cenens et al., 2013). Using this feature, phages can survive steadily and continuously in natural environments. Using different growth cycles caused by temperature differences, it is possible to use phages to control the number of Y. pestis bacteria in natural environments where the plague is present. When lysogenic bacteria are in soil and fleas, the temperature is around 21 °C, and the phage is in a pseudo-lysogenic state. When fleas bite marmots and mice, the lysogenic bacteria enter the animal's body, and the temperature increases to 37 °C. At this point, the phage enters the lysis cycle, keeping the *Y. pestis* bacteria in the body at a low concentration. If transmitted to humans, causing human plague, lysogenic bacteria can help protect the human body, reducing the severity of the illness and the risk of death.

### 4.2 Identification of phage HQ103 receptor

Currently, bacteria have developed many defense mechanisms against invading phages (Sanders and Klaenhammer, 1983). These defenses include preventing phage attachment, breaking down phage DNA inside bacteria and/or stopping replication (Goldfarb et al., 2015), starting cell death when infected (Johnson et al., 2022), and encouraging chromosome deletion through non-homologous end joining in resisting phages (Shen et al., 2018). Bacteria can lose or change phage target receptors, create a polysaccharide outer layer that stops phage attachment, or make competitive inhibitors that connect to the phage attachment site. Phages can adapt by changing where their tail fibers attach, making enzymes break down the outer layer, or altering the receptors to which they are attached (Koskella and Brockhurst, 2014). Most of the receptors found in *Y. pestis* phages are Lipopolysaccharide (LPS). For example, Filippov and others (Filippov et al., 2011) discovered that the phage receptors of eight *Y. pestis* types are the six inner and outer core parts of *Y. pestis* LPS. Moreover, Qi and others (Qi et al., 2022) reported that three P2-like phage receptors found in the Himalayas were LPS core parts of *Y. pestis*. Salem and others (Salem et al., 2021) reported that a T4-like phage found in pig waste infected Yersinia pseudotuberculosis and *Y. pestis* using LPS and OmpF as receptors. However, their article used Y. pseudotuberculosis to test for phage resistance changes, which could not prove that the *Y. pestis* receptor was also OmpF. Regarding the outer membrane protein receptor of *Y. pestis* phage, only Zhao et al. (Zhao et al., 2013) reported in 2013 that the outer membrane proteins Ail and OmpF of *Y. pestis* were involved in the attachment of T7 related phage Yep-phi. Subsequently, the OmpA receptor of *Y. pestis* has never been reported before. Our study fills this gap in the research of *Y. pestis* phage OmpA receptor and starts a new direction for *Y. pestis* phage receptor research.

Phage receptors are important for phages to enter their host (Tzipilevich et al., 2017), and a key foundation for studying phage cocktail therapy. In our study, we discovered that the outer membrane protein OmpA on the surface of *Y. pestis* is one of the receptors for phage HQ103. The base mutation of the OmpA mutant led to a change in amino acids, which altered the protein's spatial structure and affected its physical and chemical properties. Consequently, the attachment rate of the phage to Y. pestis dropped from 97% to 47%, and phage HQ103 could not lytic the OmpA mutant. The OmpA complement strain, created by introducing the OmpA gene into the OmpA mutant strain through electroporation, which could lytic again by phage HQ103. Boags et al. found that the outer membrane protein OmpA is a structure that connects peptidoglycan to the outer membrane in the cell wall (Boags et al., 2019; Paulsson et al., 2021). OmpA forms a stable structure with peptidoglycan through its C-terminal domain in a non-covalent way, and this stable structure helps maintain the position of the cell wall. Removing OmpA or partially losing its function made the mutant strain produce more vesicles than the normal strain. This might be due to the unstable position of the cell wall, and the cell wall's position could change depending on OmpA's function. OmpA is linked to peptidoglycan through the outer membrane. We think that the two amino acid mutations in OmpA make the outer membrane unable to connect stably to peptidoglycan and form a stable pathway. Consequently, the phage genome cannot enter the bacteria. This also explains why the mutant still has a 47 % attachment rate but cannot produce plaques. It is also possible that the change in OmpA's amino acid alters its function in the bacteria's outer membrane, causing most phages to fail to attach to the bacteria's surface. However, the attachment rate does not drop below 10 %, which might be because the phage's receptor is not only OmpA. LPS could also be a receptor worth studying further. By predicting the OmpA transmembrane domain model, we found two amino acid mutations, one inside the membrane and the other outside the membrane. Since the entire gene is complemented together, we speculate that only the mutant amino acid outside the membrane might play a role, leading to a lower attachment rate. In addition to investigating whether HQ103 interacts with other receptors, such as LPS, it is critical to separately complement the two mutant amino acid sites to accurately identify the specific amino acid sites that influence the receptor's function.

The widespread use of antibiotics has led to the emergence of various antibiotic-resistant bacteria, with super-drug resistant bacteria posing the most significant threat (Fisher et al., 2017). Streptomycin has long been regarded as the most effective first-line treatment for plague (Sebbane and Lemaître, 2021). While antibiotic resistance in *Y. pestis* is uncommon (Dai et al., 2021), it is important to note that the multidrug resistance plasmid pIP1202 against streptomycin has been reported (Galimand et al., 1997). The streptomycin-resistant plasmid pIP1203 (Galimand et al., 1997) and streptomycin resistance caused by mutations in the rpsL gene on chromosomes have also been identified (Dai et al., 2021). Taken together, these suggest that streptomycin, long considered the top choice for treating plague, carries a risk of treatment failure. phages can kill bacteria, may be a promising solution to this issue (Le et al., 2022). Currently, phage cocktail therapy is the most commonly used approach in clinical treatment (York, 2022; Yang et al., 2020). This method involves mixing various phages that target different receptors of host bacteria. This approach offers a valuable resource for phage therapy in treating plague.

## Nucleotide sequence number

The sequence data of phage HQ103 are available at NCBI under the accession number MZ420230

## Funding

This study was supported by the 10.13039/501100001809National Natural Science Foundation of China (NSFC) under award number 32260039.

## Author statement

Neither the entire manuscript nor any part of its content has been published or has been accepted elsewhere and this manuscript has not been submitted to any other journal. No portion of the text has been copied from other material in the literature. All of the authors in this manuscript have read and approved the final version submitted.

## CRediT authorship contribution statement

**Zijian Wang:** Methodology, Formal analysis, Data curation, Writing – original draft. **Jiao Yang:** Methodology, Validation, Formal analysis, Writing – original draft. **Lihua Yang:** Validation, Resources, Writing – review & editing. **Youhong Zhong:** Conceptualization, Resources, Funding acquisition. **Peng Wang:** Conceptualization, Writing – review & editing, Funding acquisition.

## Declaration of competing interest

The authors declare no competing financial interests.

## Data Availability

Data will be made available on request.
